# Percutaneous Isolated Hepatic Perfusion for the Treatment of Unresectable Liver Malignancies

**DOI:** 10.1007/s00270-015-1276-z

**Published:** 2015-12-30

**Authors:** Mark C. Burgmans, Eleonora M. de Leede, Christian H. Martini, Ellen Kapiteijn, Alexander L. Vahrmeijer, Arian R. van Erkel

**Affiliations:** Department of Radiology, Leiden University Medical Centre, Postal Zone C2-S, Albinusdreef 2, 2300 RC Leiden, The Netherlands; Department of Surgery, Leiden University Medical Centre, Albinusdreef 2, 2300 RC Leiden, The Netherlands; Department of Anesthesiology, Leiden University Medical Centre, Albinusdreef 2, 2300 RC Leiden, The Netherlands; Department of Medical Oncology, Leiden University Medical Centre, Albinusdreef 2, 2300 RC Leiden, The Netherlands

**Keywords:** Interventional oncology, Liver/hepatic, Percutaneous hepatic perfusion, Melphalan

## Abstract

Liver malignancies are a major burden of disease worldwide. The long-term prognosis for patients with unresectable tumors remains poor, despite advances in systemic chemotherapy, targeted agents, and minimally invasive therapies such as ablation, chemoembolization, and radioembolization. Thus, the demand for new and better treatments for malignant liver tumors remains high. Surgical isolated hepatic perfusion (IHP) has been shown to be effective in patients with various hepatic malignancies, but is complex, associated with high complication rates and not repeatable. Percutaneous isolated liver perfusion (PHP) is a novel minimally invasive, repeatable, and safer alternative to IHP. PHP is rapidly gaining interest and the number of procedures performed in Europe now exceeds 200. This review discusses the indications, technique and patient management of PHP and provides an overview of the available data.

## Introduction

The liver is frequently affected by cancer. Primary liver cancer is the sixth most common cancer in the world and the third cause of cancer-related death [1]. The liver is also a predilection site for metastases from various malignancies [1, 2]. Surgery or ablation offers the best chance of a cure in most liver malignancies, but this is often not feasible due to the extend or location of the disease. Liver malignancies have a dominant or exclusive vascular supply from the hepatic artery, whereas 70–80 % of the supply of the non-tumorous liver parenchyma is derived from the portal vein [3, 4]. This difference in perfusion is utilized in liver-directed therapies, such as trans-arterial (chemo-) embolization or radioembolization. The unique hepatic anatomy also allows vascular isolation of the liver to deliver high doses of cytotoxic agents with minimal systemic toxicity. Isolated hepatic perfusion (IHP) is a complex surgical technique that involves clamping of the inferior vena cava (IVC) and portal vein (PV), ligation of IVC tributaries and arterial hepatico-enteric anastomoses with subsequent infusion of a high dose of chemotherapy into the proper hepatic artery [5–10]. Promising results have been obtained with IHP in treating liver tumors from different histology. Response rates of 37–52 % have been reported for metastatic ocular melanoma patients [11–14]. In patients with liver metastases from colorectal carcinoma, response rates of 50–60 % have been obtained [15–17]. Despite the good response rates, the complexity and duration (up to 9 h) of the procedure have prevented for IHP to gain wide acceptance [12]. Furthermore, IHP is generally not repeatable and is associated with high morbidity and mortality rates [16, 18–20].

Percutaneous hepatic perfusion (PHP) is a novel alternative to IHP that enables vascular isolation and perfusion of the liver with the use of endovascular techniques [21]. The minimal invasiveness as well as the repeatability of PHP offers an important advantage over IHP. This review will describe this highly innovative technique and provide an update of current literature on PHP.

## Patient Selection

PHP has been performed in patients with primary tumors and various hepatic metastases (Table 1). Patients who best qualify for PHP are those who have disease of the liver only or predominantly. For obvious reasons, systemic treatment is the more appropriate therapy for patients with more extensive extrahepatic disease, if available. As significant hemodynamic perturbations occur during PHP, patients should have a normal-to-high functional capacity and have no or limited cardiopulmonary comorbidity. Although the most commonly used chemotherapeutic agent, melphalan chloride, has limited liver toxicity, patients with insufficient liver function are generally excluded from treatment. Portal hypertension, especially with concomitant hepatofugal portovenous blood flow, is a contraindication for treatment. During the procedure, adequate anti-coagulation with heparin is required to prevent intravascular thrombosis and clot formation in the extracorporeal circulation system. Therefore, patients with intolerance to heparin do not qualify for treatment nor do patients with an increased risk of bleeding (recent history of spontaneous internal bleeding or uncorrectable coagulation disorder). Women who are pre-menopausal should not be treated during the menstruation period or receive hormonal suppression therapy to prevent intra-procedural vaginal bleeding. It is generally recommended to perform computed tomography (CT) or magnetic resonance imaging (MRI) of the brain prior to treatment, as brain metastases with a propensity to bleed are a contraindication to PHP. Patients should undergo arterial phase and portovenous phase contrast-enhanced abdominal CT to confirm patency of the portal vein and screen for vascular anomalies that may render PHP difficult. Variants of the hepatic arteries are usually not a contraindication to PHP, but may require preemptive coil embolization to either redistribute hepatic flow or prevent inadvertent leakage of chemotherapeutics to the systemic circulation.

**Table 1:** Summary of conducted studies on percutaneous hepatic perfusion

References	Year	No. pts (no. PHPs)	Type of hepatic malignancy (*n*)	Chemotherapy	Type of study	Intervention	Endpoint	OR R % (*n*)	hPFS, median	OS, median	Complications^c^
Hughes et al. [53]	2015	93 (max 6 per pt)	Melanoma (ocular 83; cutaneous 10)	Melphalan	RCT	PHP^b^	Response (primary: hPFS)	27.3 % (vs 4.1 % in control)	7.0 months (vs 1.6 in controls)	10.6 months (vs 10.0 in controls)^Ω^	Neutropenia (85.7 %), thrombocytopenia (80.0 %), anemia (62.9 %), self-limiting hyperbilirubinemia (14.3 %), cardiac toxicity (12.9 %), cerebral ischemia (1.2 %), death 3.2 %
Vogl et al. [46]	2014	14 (18)	Melanoma (ocular 8; cutaneous 3), Gastric/Breast/CA (1)	Melphalan	Retrospective	PHP^b^	Response and toxicity	54 (7)	n.r.	n.r.	Pancytopenia; death (7.1 %; retroperitoneal hemorrhage)
Fitzpatrick et al. [57]	2014	5 (15)	Melanoma (ocular 4; cutaneous 1)	Melphalan	Case series	PHP^b^	Feasibility and toxicity	n.r.	n.r.	n.r.	Transient mild hypothermia and metabolic acidosis
Fukumoto et al. [42]	2014	68 (103)	HCC, BCLC intermediate (27) or advanced (41)	Mitomycin C and/or doxorubicin	Prospective	Resection + PHP	Response	70.6 (48)	n.r.	25 months	Leukopenia (44.1 %), serum AST gr. 3/4 (77.9 %), hair loss (72 %), gastroduodenal ulcer (4.4 %)
Forster et al. [52]	2013	10 (27)	Melanoma (ocular 5; cutaneous 3, unknown 1). Sarcoma (1)	Melphalan	Retrospective	PHP^b^	Response and toxicity	50 (5)	240 days	n.r.	Bone marrow suppression; mild elevation serum troponin (70 %)
Pingpank et al. [58]	2011^a^	23 (68)	NET (23)	Melphalan	Prospective	PHP^b^	Response (ORR)	79	39 months	n.r.	Acute transaminitis gr 3/4 (22 %); neutropenia 47 %, thrombocytopenia 29 %, anemia (15 %). Death 0.04 % (cholangitis)
Miao et al. [30]	2008	51 (136)	Melanoma (ocular 12, cutaneous 4), NET (12), CRC (7), HCC (5), RCC (4), AdrC/Breast/CA (2), Ewing (1)	Melphalan	Prospective	PHP^b^	Hemodynamics and metabolic changes	n.r.	n.r.	n.r.	Transient hypotension and metabolic acidosis; nausea/vomiting (10 %)
Pingpank [39]	2005	28 (74)	Melanoma (ocular 10, cutaneous 3), CRC (2), Hepatobiliary (3), NET (4), RCC (2), AdrC/Breast/Sarcoma/PA (1)	Melphalan	Phase I	PHP^b^	MTD, toxicity, pharmacokinetics	29.6 (8)	n.r.	n.r.	Neutropenia gr 3/4 (73.6 %); thrombocytopenia gr 3/4 (36.8 %); anemia (21.1 %)^e^
Ku et al. [59]	2004	22 (40)	HCC (22)	Doxorubicin	Prospective	resection + PHP	Efficacy	86 (19)	n.r.	1 and 5 years OS: 86 and 47 %	Leukopenia (45.5 %), hair loss (63.6 %)
Savier et al. [48]	2003	4 (10)	Breast/CRC/Gastric/CA (1)	Melphalan	Prospective study	IHP + PHP	Feasibility; pharmacokinetics	n.r.	n.r.	n.r.	Neutropenia grade 3/4 (50 %)
Ku et al. [56]	1998	28 (39)	HCC, TNM III (1) or IV-A (27)	Doxorubicin	Prospective	PHP	Response and survival	63 (17)	n.r.	16 months	Chemical hepatitis (71 %), leukopenia (54 %), hair loss (43 %), thrombocytopenia (18 %), hemolysis/hematuria (57 %), gastroduodenal ulcer (7 %), death 8 % due to pancreatitis (4 %) and HAT (4 %)
Ku et al. [60]	1997	16 (16)	HCC (11), CRC (1), Breast CA (1), Melanoma (1)	Doxorubicin	Prospective	PHP	Hemodynamics, pharmacology, toxicity	n.r.	n.r.	n.r.	Chemical hepatitis (75 %), leukopenia (43.7 %), alopecia (37.5 %), thrombocytopenia (25 %), hemolysis/hematuria (50 %)
Ku et al. [40]	1995	15 (15)	HCC, unresectable (15)	Doxorubicin	Phase I	PHP	Hemodynamics, pharmacology, toxicity, response	64 (9)	n.r.	12 months for responders (vs 5 for non-responders)	Chemical hepatitis (71 %), leukopenia (67 %), alopecia (33 %), thrombocytopenia (40 %), hemolysis/hematuria (87 %), gastroduodenal ulcer (14 %), death 13.3 % due to pancreatitis (7 %) and HAT (7 %)
Ravikumar et al. [29]	1994	21 (58)	HCC (5), CRC (8), Melanoma (2), Sarcoma (4), Adrenal/Pancreatic/SCLC/CA (*n* = 1)	5-FU, doxorubicin	Phase I	PHP	MTD, feasibility	9.5 (2)	n.r.	n.r.	Hematologic, primarily leukopenia/neutropenia; transient hypotension (78.5 %)

*ORR* objective response rate (complete plus partial response), *NET* neuroendocrine tumor, *HCC* hepatocellular carcinoma, *CRC* colorectal carcinoma, *RCC* renal cell carcinoma, *AdrC* adrenalcortical carcinoma, *CA* cholangiocarcinoma, *SCLC* small cell lung carcinoma, *PA* periampullary carcinoma, *BCLC* barcelona clinic liver criteria, *n.r.* not reported, *hPFS* hepatic progression-free survival, *MTD* maximum tolerated dose, *HAT* hepatic artery thrombosis

^a^Only presented as an abstract

^b^Delcath system

^c^List is not extensive, mortality and common complications are reported

^d^Not reported in the abstract

^e^Complication rates are quoted per PHP at MTD

^Ω^57.1 % of patients in control group crossed over to PHP

## Hepatic Vascular Mapping

**Fig. 1:**
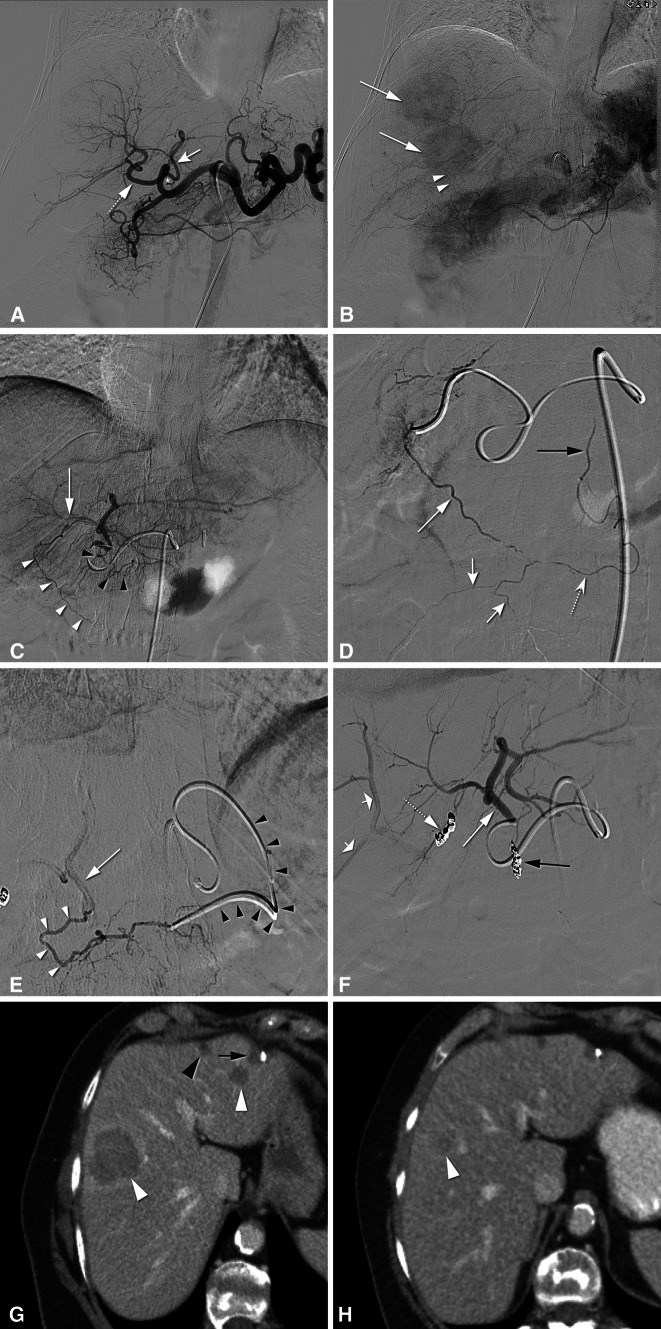
Hepatic vascular mapping in a 63-year-old female with bilateral hepatic metastases from ocular melanoma. **A** Angiographic images from the celiac trunk show a right gastric artery (*asterisk*) originating from the left hepatic artery (*white arrow*). **B** In the late arterial phase, two hypervascular metastases in the right liver lobe (*white arrows*) are seen as well as the falciform artery (*arrowheads*). A treatment plan was made to perform PHP with selective infusion of melphalan chloride into the left hepatic artery (LHA) and right hepatic artery (*dotted arrow* in **A**) with preemptive coiling of the right gastric (RGA) and falciform artery (FA). **C** Selective angiography from the LHA shows the FA (*white arrowheads*) to be originating from the segment four artery (*white arrows*). The RGA is also depicted (*black arrowheads*). **D** Selective angiography of the FA (*long white arrow*) shows opacification of the right internal thoracic artery (*black arrow*) through the ensiform artery (*dotted arrow*) and of anterior abdominal wall arteries (*short white arrows*). **E** Antegrade catheterization of the RGA was unsuccessful and therefore, retrograde catheterization was performed via the left gastric artery (LGA) using a 2.4-F microcatheter (*black arrowheads*). Angiography shows the RGA (*white arrowheads*) and LHA (*white arrow*). **F** Angiography of the LHA (*white arrow*) after successful coiling of the FA (*dotted arrow*) and RGA (*black arrow*) with 2-mm detachable microcoils. Some reflux of contrast is seen in right hepatic artery branches (*short white arrows*). **G** Axial CT image in portovenous phase before treatment demonstrates two hepatic metastases (*white arrowheads*). A third metastasis was seen in segment 4B (not shown). In the left liver lobe a cyst is seen (*black arrowhead*) as well as a hypodensity caused by previous laparoscopic excisional biopsy (*black arrow*). **H** CT in portovenous phase after two cycles of PHP shows marked reduction in size of the right liver lobe metastasis. The other two metastases showed a complete radiological response after treatment

Prior to PHP, angiography of the celiac trunk and hepatic arteries should be performed to delineate the arterial supply of the liver (Fig. 1). Angiography of the superior mesenteric artery may provide additional information and is always performed in patients with an aberrant right hepatic artery or to obtain an indirect portogram if hepatofugal or compromised portovenous flow is suspected. After mapping of the hepatic arterial circulation, a strategy for chemotherapy infusion is formulated. Infusion into the common or proper hepatic artery allows whole-liver treatment without repositioning of the catheter, but carries a higher risk of inadvertent flow of chemotherapeutic drugs into branches with supply to the gastrointestinal tract. To prevent this, coil embolization may be indicated of arteries at risk, such as the gastroduodenal and right gastric artery. The use of cone-beam CT is recommended as this improves the detection of vascular variants, extrahepatic enhancement, and extrahepatic vascular tumor supply [22–26]. Embolization of aberrant hepatic arteries has been proven to be an effective strategy to redistribute flow in transarterial liver therapies such as radioembolization [27]. Hepatic vascular mapping is generally performed several days to a week prior to PHP.

## PHP Procedure

At present, only one PHP system is commercially available (Chemosaturation Hepatic Delivery System, Delcath Systems Inc, New York, USA), and therefore, some of the techniques described are specific to this system. In Japan, another double-balloon catheter (4L/2B, Fuji System Co. Ltd, Tokyo, Japan) is currently used in clinical studies.

**Fig. 2:**
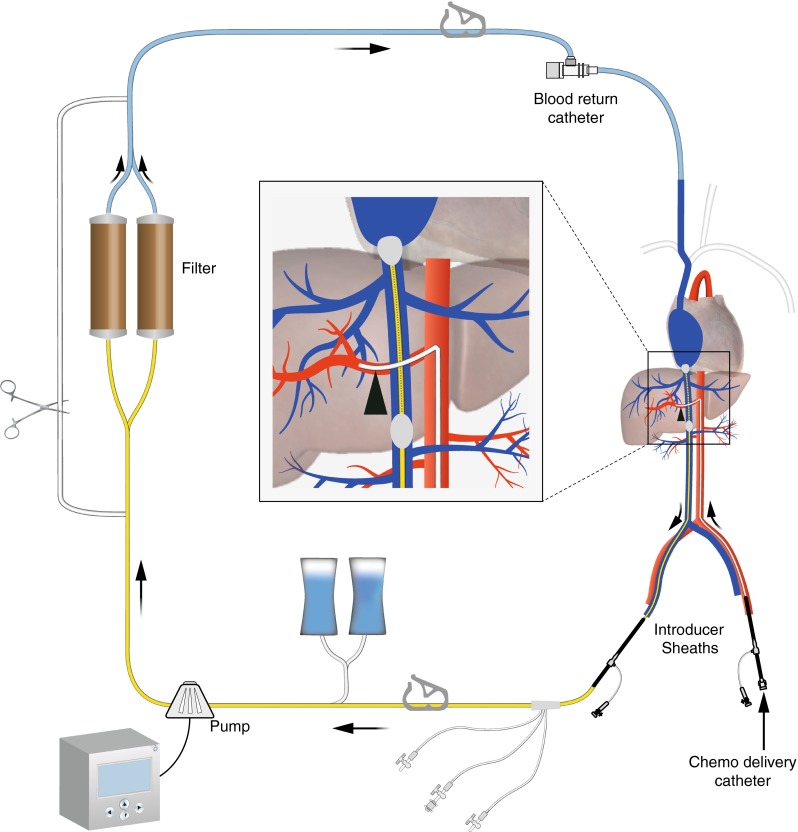
Schematic display of the setup of percutaneous hepatic perfusion. Chemotherapeutic drugs are infused through a catheter placed in the hepatic artery (*arrowhead*) and the effluent chemosaturated blood returning through the hepatic veins is aspirated through the side holes of the double-balloon catheter. An extracorporeal system with carbon activated filters is used to separate the chemotherapeutics from the blood, before the blood is returned through a sheath in the internal jugular vein

**Fig. 3:**
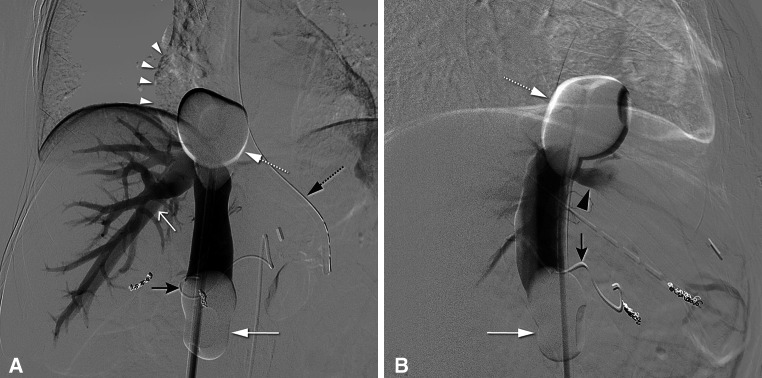
Same patient as in Fig. 1. Postero-anterior (**A**) and lateral (**B**) images during venography performed by hand injection of non-diluted contrast medium through the size holes of the double-balloon catheter. The cranial balloon (*dotted white arrow*) was positioned at the atriocaval junction to prevent flow to the right atrium (*white arrowheads*). The caudal balloon (*white arrow*) prevented retrograde flow to the infrarenal inferior vena cava. A microcatheter (*black arrow*) was placed through a 5-F celiac catheter and into the left hepatic artery for the infusion of melphalan chloride. Both the right hepatic vein (*open white arrow*) and middle hepatic vein (*black arrowhead*) were opacified. Also, a nasogastric tube is seen (*dotted black arrow*)

The procedure is performed under general anesthesia by a team consisting of a dedicated interventional radiologist, anesthesiologist, and an extracorporeal perfusionist. A cannula is placed in the radial artery for continuous arterial pressure monitoring, and a urinary bladder catheter inserted. A triple-lumen line is placed in the left internal jugular vein (IJV) for central venous pressure monitoring and infusion of sympathomimetics and fluids. Access to the right IJV is created with a 10-F vascular sheath, to the right common femoral vein (CFV) with an 18-F sheath and to the left common femoral artery (CFA) with a 5-F sheath (see Fig. 2). After all lines and sheaths have been placed, heparin is administered at an initial dose of 300 U/kg, and the activated clotting time (ACT) is maintained above 400 s during the entire procedure. Hepatic angiograms are obtained, and the tip of a microcatheter is then placed into the hepatic artery at the intended location of infusion. In a selective lobar approach, the dose of chemotherapy is split and infused into the right and left hepatic artery separately. In most patients, the ratio of the two lobes is such that 60 % of total dose is to be injected into the right hepatic artery and 40 % into the left hepatic artery. The disadvantage of consecutive lobar infusions is that it prolongs the time of extracorporeal circulation, as the chemotherapy infusion has to be interrupted to reposition the catheter. After placement of the infusion catheter in the hepatic artery, a 16-F double-balloon catheter (Isofuse Isolation Aspiration Catheter, Delcath Systems Inc, New York, NY, USA) is inserted via the right CFV and positioned with its tip in the right atrium. The catheter is then connected to an extracorporeal circulation system consisting of a centrifugal pump and two drug filtration activated carbon filters. Blood is aspirated through catheter fenestrations in a segment between the two balloons, actively pumped through the filtration system and returned through the sheath in the IJV. The cranial balloon of the catheter is then inflated in the right atrium and retracted into the inferior caval vein (ICV) until the shape of the balloon resembles that of an acorn. The caudal balloon is inflated in the IVC below the level of the hepatic veins and above the level of the renal veins. With both balloons inflated, a venogram is obtained by hand injection of a contrast medium through the injection port of the double-balloon catheter (Fig. 3). With adequate positioning of the double-balloon catheter, flow of the effluent hepatovenous blood back to the systemic circulation is prevented by the cranial balloon at the atriocaval junction and by the caudal balloon at the level of the retrohepatic ICV.

Once correct positioning of the two balloons is confirmed, a stepwise approach is used to start filtration of blood by the two cartridges. A centrifugal pump is used to achieve a flow rate between 0.40 and 0.75 L/min. The maximal flow rate should not exceed 0.8 L/min and pre-pump pressures should not exceed −250 mmHg to avoid the catheter to collapse or kink. The hemofiltration filters are brought online one by one, by removing the clamps. Once the cartridges are completely filled with blood, the bypass line is closed. When the hemofiltration circuit is running sufficiently and hemodynamic stability is achieved (see below), intra-arterial infusion of chemotherapeutic drugs may be started using a pump injector and a flow rate of 0.4 mL/s. Before and during the infusion, hepatic angiograms are obtained to ensure that hepatic blood flow is not compromised. If the angiograms show arterial spasms, this may be treated with nitroglycerine boluses of 100–200 micrograms. After the infusion, extracorporeal filtration is continued for 30 min (‘washout period’) to allow clearance of chemotherapeutics from the liver. At the end of the procedure, the effects of heparin are reversed by administration of protamine sulfate on a 1:1 basis (1 mg of protamine sulfate to antagonize 1 mg of heparine). The vascular sheaths are left in place until coagulation is sufficiently corrected, although a vascular closure device may be placed immediately after the procedure to achieve hemostasis at the arterial puncture site. The duration of the procedure is generally 3–4 h.

## Anesthesiology and Perfusionist Support

PHP is associated with hemodynamic and metabolic changes that require monitoring and management by an experienced anesthesiologist. In early studies, the conduct of PHP under local anesthesia and sedation has been described, but nowadays procedures are generally performed under general anesthesia [28, 29]. PHP results in significant decreases in mean arterial and central venous pressures and increases in heart rate compared to baseline [29, 30]. Decreases in blood pressures generally occur at two stages: upon occlusion of the IVC and when blood flow is diverted through the filters. Inflation of the balloons of the double-balloon catheter results in a decrease in central venous return and right atrium pre-load. This first drop in blood pressure is corrected by administration of fluids and norepinephrine and/or phenylephrine to maintain a mean arterial pressure above 60 mmHg. A second drop in blood pressure may be attributed to the depletion of sympathomimetics by the activated carbon filters. Infusion rates as high as 0.2–1.5 μg/kg/min for norepinephrine and 0.4–3.0 μg/kg/min for phenylephrine are generally required during the perfusion period as 67–95 % of the sympathomimetics are cleared from the blood by the filters [29]. A decrease in patient’s body temperature is also commonly encountered during PHP and is a result of the blood flowing through the non-heated extracorporeal circuit. In general, the hypothermia is not severe and may be reduced by using an air-warming system [30].

## Chemotherapeutic Agent

The majority of studies have used melphalan chloride as the chemotherapeutic agent of choice, for it has pharmacological properties to make it suitable for PHP. It can easily be administered intra-arterially, has limited liver toxicity, a high hepatic extraction rate, a very short half-life, and an immediate effect on tumor cells [31, 32]. The currently available filtration system (Delcath hemofiltration cartridges) is specific for melphalan chloride.

Melphalan chloride is an alkylating agent of the nitrogen mustard group. Its binding to deoxyribonucleic acid (DNA) can result in cross-linking between bases on complementary strands leading to double-stranded DNA breaks and eventually cell death [8, 10, 33–38]. In a phase I dose-escalating study with percutaneous administration, the maximum tolerated dose (MTD) of melphalan chloride was 3.0 mg/kg body weight [39]. The maximum total dose is generally limited to 220 mg. Because of the short life of melphalan chloride, the drug should be prepared in the pharmacy just prior to administration.

Several studies have used doxorubicin as the chemotherapeutic agent, mainly in patients with hepatocellular carcinoma (HCC) [29, 40–43]. Doxorubicin has some disadvantages over melphalan chloride as an agent for PHP. Firstly, it has a first-pass hepatic extraction fraction that is only around 60 % [44]. Secondly, doxorubicin is associated with considerable liver toxicity. Studies on PHP with doxorubicin have reported chemical hepatitis rates of >70 % [29, 40–43]. The chemical hepatitis was generally mild to moderate and self-limiting. Thirdly, with the currently available filters, the doxorubicin infusion time, and consequently the administrated dose, is limited as prolonged infusion may lead to increased systemic exposure. In a study by Ku et al., a mean doxorubicin extraction rate of 91 % was found, but the filtration rated dropped to 55 % at 20 min [40]. Nevertheless, very promising results have been obtained with doxorubicin in patients with advanced HCC with PHP as either the primary treatment or as an adjunct to surgery (see results section). The hemofiltration cartridges (DHP-1; Kuraray Co., Ltd., Osaka, Japan) used in this study differ from those in studies with melphalan chloride.

## Post-procedural Care

Patients are monitored in a medium or intensive care unit (ICU) 12–24 h after the procedure and are generally discharged after 2–3 days. This compares favorably to IHP for which admission to the ICU is generally several days and a mean hospital stay has been reported of 10–29 days [45]. Anemia, neutropenia, and thrombocytopenia may be seen early after the procedure and may (in part) reflect dilution as a result of peri-procedural fluid administration. Transfusion with fresh-frozen plasma, packed red blood cells, or platelets may occasionally be needed. PHP is associated with transient metabolic acidosis, but infrequently to such a degree that correction with sodium bicarbonate is required [32].

## Complications

Table 1 provides an overview of the type and frequency of common and serious complications as reported in the literature. The most common adverse effects result from bone marrow suppression leading to neutropenia, thrombocytopenia, and/or anemia. Mild-to-moderate bone marrow suppression is seen in half to three-quarters of patients. The nadir of cytopenia is generally 10–14 days after PHP. It is generally recommended to administer granulocyte colony-stimulating factor analogues (pegfilgastrim) within 48 h after PHP to anticipate bone marrow depression. Symptomatic anemia and severe thrombocytopenia (<20,000/mm^3^) may require transfusions. Regular blood tests in the first 2 weeks after PHP are recommended.

Complications related to multiple vascular accesses and vessel catheterization might occur. Patients are at an increased risk of puncture site bleeding as high doses of heparin are administrated during the procedure to prevent clot formation in the extracorporeal circuit. Bleeding other than from the puncture site is uncommon, but may have severe consequences [29, 30, 46]. The hypotension associated with PHP may potentially result in complications such as organ ischemia. In general, the hypotension is of short duration and responds well to administration of fluids and sympathomimetics.

The reported mortality rate of PHP is 0–13.3 % (reference Table 1). Most of the published deaths occurred in early studies at the beginning of the learning curve and with a system that was different from the kit that is currently used. The reported IHP-related death rate is much higher than that of PHP: 5–27 % [45].

## Leakage to the Systemic Circulation

Bone marrow depression is a result of leakage of melphalan chloride to the systemic circulation. Increased systemic exposure to melphalan chloride may be a result of incomplete filtration of the chemotherapeutic agent by the hemofiltration filters. In a phase I dose-escalating study, pharmacological blood samples were obtained during 74 procedures in 28 patients with unresectable hepatic malignancies [38]. Perfusions were performed with Hemosorba drug filtration cartridges (Asahi Medical Co, Tokyo, Japan) for which the filter extraction percentages ranged from 58.2 to 94.7 %, with a mean of 77 %. A second-generation filter system is available since 2012, and this filter was reported to have an efficiency rate of 99 % in preclinical studies [47]. Initial experiences with the second-generation filter seem to indicate that the degree of bone marrow depression with this filter is lower compared with that associated with previous filter systems [46].

There may be causes of melphalan chloride leakage other than through the filter system. The fact that, even with the second-generation filter, mild-to-moderate bone marrow depression is not infrequently seen seems to suggest that leakage other than through the hemofiltration system indeed occurs [46]. One potential cause of leakage may be insufficient sealing of the balloon at the atriocaval junction with consequent leakage alongside the balloon. Furthermore, leakage to the systemic circulation could also be a result of the presence of collateral pathways between the IVC and azygos, hemiazygos, accessory hemiazygos, thoracolumbar, and/or diaphragmatic veins. Small interconnecting veins between the aforementioned structures are not uncommon and may cause the melphalan chloride to bypass the extracorporeal filter system. Another possible mechanism may be the uptake of melphalan chloride by the hepatobiliary system and storage until the balloons are deflated, after which melphalan chloride is released systemically. However, IHP is associated with lower rates of leakage of chemotherapeutic drugs than PHP [48]. Furthermore, the half-life of melphalan chloride is very short. It therefore seems less plausible that post-procedural release of chemotherapeutics by the liver is the cause of systemic toxicity.

In IHP, leakage can be monitored by injection of human serum albumin (HSA) or erythrocytes labelled with iodine-31 or technetium-99 [45, 49, 50]. A closed, recirculating system is used in IHP, and detection of labelled HSA or erythrocytes in the systemic circulation is an indication of leakage. Unfortunately, this method cannot be applied in PHP as the perfusion circuit is not a closed system, and the activated carbon filters allow passage of both HSA and erythrocytes. Leakage can be quantified by measurement of systemic drug levels during PHP, but this does not provide real-time information as laboratory tests to determine melphalan plasma levels are rather complex and time-consuming.

## Results of PHP to Date

**Fig. 4:**
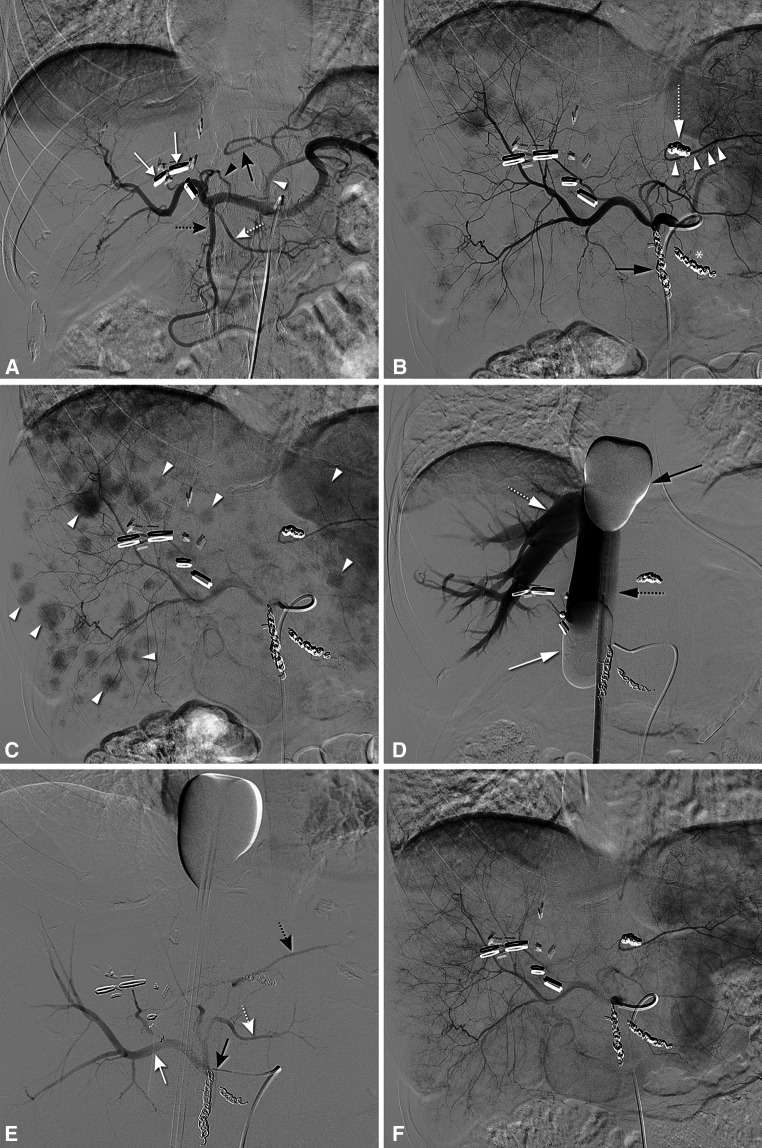
Hepatic vascular mapping and PHP in a 44-year-old female with bilateral hepatic metastases from ocular melanoma. **A** Angiography from the celiac trunk showed the gastroduodenal artery (GDA) (*dotted black arrow*), the right gastric artery (RGA) (*dotted white arrow*), a segment 3 artery (S3) (*black arrowhead*) originating from the left hepatic artery and a segment 2 artery (S2) (*black arrow*) originating from the left gastric artery (*white arrowhead*). **B** After coil embolization of the GDA (*black arrow*), RGA (*asterisk*) and aberrant left hepatic artery (*open white arrow*), redistribution of flow to S2 (*arrowheads*) was established through intrahepatic collaterals. **C** Multiple hypervascular tumors are seen in both lobes (*arrowheads*). **D** After inflation of the cranial (*black arrow*) and caudal (*white arrow*) balloon of the double-balloon catheter, the inferior caval vein (*open black arrow*) and right hepatic vein (*white open arrow*) were opacified during venography. No leakage was demonstrated alongside the balloons. **E** Angiography with a microcatheter positioned in the proper hepatic artery showed opacification of all hepatic arteries, including the right hepatic artery (*white arrow*), S2 (*open black arrow*), and S3 (*open white arrow*), just prior to infusion of melphalan chloride. **F** At the start of the *second* PHP 6 weeks later, angiography shows complete disappearance of staining of the liver metastases. Follow-up CT and PET/CT (not shown) after two PHP procedures revealed three small residual tumors that were subsequently treated with RFA. The patient remained without evidence of disease until 1 year after the first PHP

The data of the efficacy of PHP are limited, and only one randomized controlled trial has been published to date. Table 1 provides an overview of PHP studies, excluding case reports and small case series. The number of procedures per patients varies from 1 to 3 between the different studies as the optimal treatment schedule and indications for retreatment have not yet been established. Most studies on PHP have been conducted in patients with liver metastases from ocular melanoma (see Fig. 4). Patients with liver metastases from ocular melanoma are evident candidates for liver-directed locoregional therapy because of the remarkable metastatic pattern of this tumor. Metastases occur in approximately 50 % of patients with ocular melanoma. In those patients with metastases, the liver is affected in 95 % of patients, and in 80 % of patients the disease has only spread to the liver [2, 51]. Ocular melanoma has a high sensitivity to melphalan chloride, and there are currently no systemic therapies with proven long-term efficacy for this tumor type.

In 2005, Pingpank et al. [39] published results of a phase I dose escalation study on PHP with melphalan chloride in 28 patients with primary and metastatic hepatic disease, establishing a MTD of 3 mg/kg. Response and survival rates were not primary endpoints, but were reported. In the 10 patients with metastases from ocular melanoma, an objective response rate (ORR) of 50 % was observed: two complete responses (CR) and three partial responses (PR). In the total study group, six PRs were documented (21.4 %). The duration of CR was 10 and 12 months, and duration of PR included two patients with ongoing responses at 9 and 11 months.

In a retrospective study by Forster et al., including 10 patients with hepatic metastases from ocular melanoma (*n* = 5), cutaneous melanoma (*n* = 3), melanoma from unknown origin (*n* = 1), or sarcoma (*n* = 1), nine patients (90 %) had stable disease or PR on follow-up imaging [52]. The median percent decrease in hepatic tumor volume was 48.6 % for patients with ocular melanoma compared to 33.3 % for the entire cohort. At a median follow-up of 11.5 months (range 4–55 months), median hepatic-free survival was 240 days and median overall survival from the time of first PHP was 8.7 months.

A retrospective two-center study reported the results of PHP in 14 patients treated with 18 PHP procedures [46]. The majority of patients (*n* = 11; 78.5 %) had liver metastases from melanoma [ocular (*n* = 8) or cutaneous (*n* = 3)]. A 50 % ORR was reported (one CR in a patient with cholangiocarcinoma and six PRs in patients with metastases from melanoma) and 38 % patients had stable disease.

Recently, the results were published of a multi-center, randomized controlled study comparing PHP with best alternative care (BAC) in patients with hepatic metastases from melanoma [53]. The study included 93 patients with unresectable hepatic metastases from either ocular (*n* = 83) or cutaneous (*n* = 10) melanoma. Patients with limited extrahepatic disease were allowed to enter the study, although most patients (59.1 %) had metastases confined to the liver. Patients in the PHP arm (*n* = 44) underwent a maximum of six isolated liver perfusions with melphalan at 4–8 weekly intervals. Patients in the control group (*n* = 49) received best alternative care (BAC) with the majority of patients (81.6 %) receiving active treatment such a systemic chemotherapy, chemoembolization, radioembolization, and surgery. A statistically significant improvement in hepatic progression-free survival (hPFS) and overall progression-free survival (oPFS) was demonstrated in patients treated with PHP compared to BAC. The hPFS and oPFS were 7.0 and 5.4 months respectively for the PHP group compared to 1.6 and 1.6 months respectively for the BAC group (*p* < 0.0001). No statistically significant difference in overall survival (OS) was found between the PHP and BAC group (10.6 and 10.0 months respectively), but this was confounded by a large proportion of the patients in the BAC group (57.1 %) crossing over to receive PHP after progression of disease.

A Japanese group has published several studies on PHP in patients with HCC using a different double-balloon catheter and hemofiltration system (see above). Some overlap between the patient groups in the different studies exists [42, 54–56]. In a prospective study, 28 patients with advanced HCC (TNM III or IV-A) underwent an average of 1.4 PHP with doxorubicin. The ORR was 63 % and the OS was 16 months. The 1-, 3-, and 5-year survival rates were 67.5, 39.7, and 39.7 %, respectively [41]. In a recent publication, the results were reported of combined reductive surgery and PHP with mitomycin C and/or doxorubicin in 68 patients with intermediate- or advanced-stage HCC [42]. An ORR of 70.6 % was achieved with a median OS of 25 months.

## Future Directions

PHP holds promise as a locoregional therapy for patients with hepatic malignancies. The ability to deliver high doses of chemotherapy with limited systemic exposure is appealing, and the minimally invasive nature of the procedure offers great advantages over IHP. In phase I studies, the feasibility and toxicity profile of the procedure have been well established. Data on the efficacy of PHP are limited, but initial results are promising, especially in the treatment of liver metastases from ocular melanoma. A recent phase III trial showed superiority of PHP over BAC in patients with hepatic metastases from either ocular (89.2 %) or cutaneous (10.8 %) melanoma [53]. Further studies are needed to establish the role of PHP in the treatment of different types of hepatic malignancies, define the optimal treatment frequency and interval, and compare treatment outcomes with currently available locoregional and systemic therapies. Prospective studies on the efficacy of PHP for primary liver tumors as well as hepatic metastases from various origins are currently being conducted (see Table 2). The most frequent toxicity associated with PHP is bone marrow depression as a result of leakage of melphalan chloride. With the introduction of the second-generation hemofiltration system, the rate and severity of bone marrow depression appear to be reduced. To further reduce the systemic toxicity rates, more studies are needed to analyze the causes of systemic leakage of chemotherapeutics and improve the technique and hemofiltration system of PHP. Currently, melphalan chloride is the most commonly chemotherapeutic agent used for PHP. In studies using either fluorouracil or doxorubicin, the affinity of the filters used was either limited or the extraction rated dropped after prolonged chemotherapy infusion [29, 40]. New detoxification filters factory tuned to high affinity for specific chemotherapeutics may enable more effective treatment with other drugs than melphalan chloride in the future.

**Table 2:** Summary of ongoing prospective studies on percutaneous hepatic perfusion

Study design	Type of hepatic malignancy	Initiation	Treatment	Estimated enrollment	End-points	Status
Phase II, single center	Ocular melanoma	Investigator^a^	PHP with melphalan. 2 cycles	20	ORR, post-PHP resectability safety, OS, HPFS, PFS, QoL	Recruiting
Phase III, multicenter	Ocular melanoma	Industry^b^	PHP with melphalan. Max 6 cycles versus BAC (dacarbazine, TACE, ipilimumab or pembrolizumab)	240	OS, PFS, ORR, HPFS, hepatic ORR, QoL	Launching fourth quarter 2015
Phase II, single center	Colorectal carcinoma	Investigator^a^	PHP with melphalan. 2 cycles	34	ORR, post-PHP resectability safety, OS, HPFS, PFS, QoL	Recruiting
Phase II, multicenter	HCC or ICC	Industry^b^	PHP with melphalan. 2 Cycles	42	ORR, safety, PFS	Recruiting
Phase II, multicenter	HCC	Industry^b^	PHP with melphalan. 3 cycles, followed by sorafenib	31	Adverse events, ORR, PFS, pharmacokinetics, QoL	Recruiting
Phase I/II	HCC	Investigator^c^	PHP followed by sorafenib	30	PFS, OS, safety	Recruiting

*HCC* hepatocellular carcinoma, *ICC* intrahepatic cholangiocarcinoma, *BAC* best alternative care, *TACE* transarterial chemoembolization, *ORR* objective response rate, *OS* overall survival, *HPFS* hepatic progression-free survival, *PFS* progression-free survival, *QoL* quality of life

^a^Leiden University Medical Center, the Netherlands

^b^Delcath Systems Inc. USA

^c^Kobe University, Japan

In conclusion, PHP is a novel, minimally invasive, and repeatable alternative to IHP. Phase I studies have demonstrated PHP to be feasible and safe. A recently published randomized controlled trial has shown improved control of liver disease compared to standard available therapy in patients with hepatic metastases from (ocular) melanoma. Further phase II and III studies are needed to define the role of PHP in the clinical management of patients with different hepatic malignancies.
